# Aerosol exposure of live bird market workers to viable influenza A/H5N1 and A/H9N2 viruses, Cambodia

**DOI:** 10.1111/zph.13009

**Published:** 2022-11-21

**Authors:** Paul F. Horwood, Srey Viseth Horm, Sokhoun Yann, Songha Tok, Malen Chan, Annika Suttie, Phalla Y, Sareth Rith, Jurre Y. Siegers, Sorn San, Holl Davun, Sothyra Tum, Sowath Ly, Arnaud Tarantola, Philippe Dussart, Erik A. Karlsson

**Affiliations:** ^1^ Virology Unit Institut Pasteur du Cambodge, Pasteur Network Phnom Penh Cambodia; ^2^ College of Public Health, Medical and Veterinary Sciences James Cook University Townsville Queensland Australia; ^3^ Epidemiology and Public Health Unit, Institut Pasteur du Cambodge Pasteur Network Phnom Penh Cambodia; ^4^ School of Applied and Biomedical Sciences Federation University Australia Churchill Victoria Australia; ^5^ National Animal Health and Production Research Institute, Cambodian Ministry of Agriculture, Forestry and Fisheries Phnom Penh Cambodia; ^6^ Present address: Regional Epidemiology Unit Santé Publique France Paris France; ^7^ Present address: Institut Pasteur de Madagascar Pasteur Network Antananarivo Madagascar

**Keywords:** A/H5N1, A/H9N2, aerosol, avian, influenza, live bird market

## Abstract

Live bird markets (LBMs) have been identified as key factors in the spread, persistence and evolution of avian influenza viruses (AIVs). In addition, these settings have been associated with human infections with AIVs of pandemic concern. Exposure to aerosolised AIVs by workers in a Cambodian LBM was assessed using aerosol impact samplers. LBM vendors were asked to wear an air sampler for 30 min per day for 1 week while continuing their usual activities in the LBM during a period of high AIV circulation (February) and a period of low circulation (May). During the period of high circulation, AIV RNA was detected from 100% of the air samplers using molecular methods and viable AIV (A/H5N1 and/or A/H9N2) was isolated from 50% of air samplers following inoculation into embryonated chicken eggs. In contrast, AIV was not detected by molecular methods or successfully isolated during the period of low circulation. This study demonstrates the increased risk of aerosol exposure of LBM workers to AIVs during periods of high circulation and highlights the need for interventions during these high‐risk periods. Novel approaches, such as environmental sampling, should be further explored at key high‐risk interfaces as a potentially cost‐effective alternative for monitoring pandemic threats.

**Impacts**

- We determined that avian influenza viruses of public health importance (A/H5N1 and A/H9N2) could be detected in the air column of a live bird market during a period of known high circulation.
- Testing showed that many of the viruses detected in the study were infectious, thus highlighting the risk of aerosol exposure to avian influenza viruses in live bird markets.
- Further research is needed to better understand the routes of exposure for zoonotic pathogens at high‐risk interfaces and to determine if interventions during periods of high circulation can reduce the risk of zoonotic transmission.

## 1 INTRODUCTION

Live bird markets (LBMs) are major hubs for poultry commerce throughout the world. Commonly, a variety of birds enter LBMs through the poultry value chain, including from backyard and semi‐commercial producers. Close contact between birds of many species from different sources leads to infection and amplification of numerous pathogens, including avian influenza viruses (AIVs). These pathogens then persist in the LBM environment due to insufficient biosecurity measures. As such, LBMs provide numerous interfaces for human exposure to AIVs, including contact with the contaminated market environment, direct contact with poultry or poultry products, and inhalation of viral particles suspended in the air column (Amonsin et al., 2008; Indriani et al., 2010; Vergne et al., 2019; Wang et al., 2006). Most LBM surveillance systems focus on detection of viruses in the birds themselves. However, it is more likely that LBM workers will be infected by aerosolized viruses in the market environment. Limited studies have been conducted to investigate this route of exposure including comparing environmental/animal samples collected concurrently and studies to estimate the risk of human infection (Bui et al., 2019; Zhou et al., 2016).

Previous longitudinal studies in Cambodian LBMs have documented a consistent pattern of AIV circulation, with peak viral activity occurring during festival periods, especially between January–March and October–November. The period of lowest circulation occurs at midyear (Horm et al., 2011, 2016; Horwood et al., 2018; Karlsson et al., 2019). To examine how AIV circulation in poultry compared with the presence of AIVs in the LBM air column and assess the risk of LBM workers inhaling live virus during their routine work activities, we collected concurrent poultry, wash water and air samples during periods of high and low viral circulation.

## 2 MATERIALS AND METHODS

**FIGURE 1:**
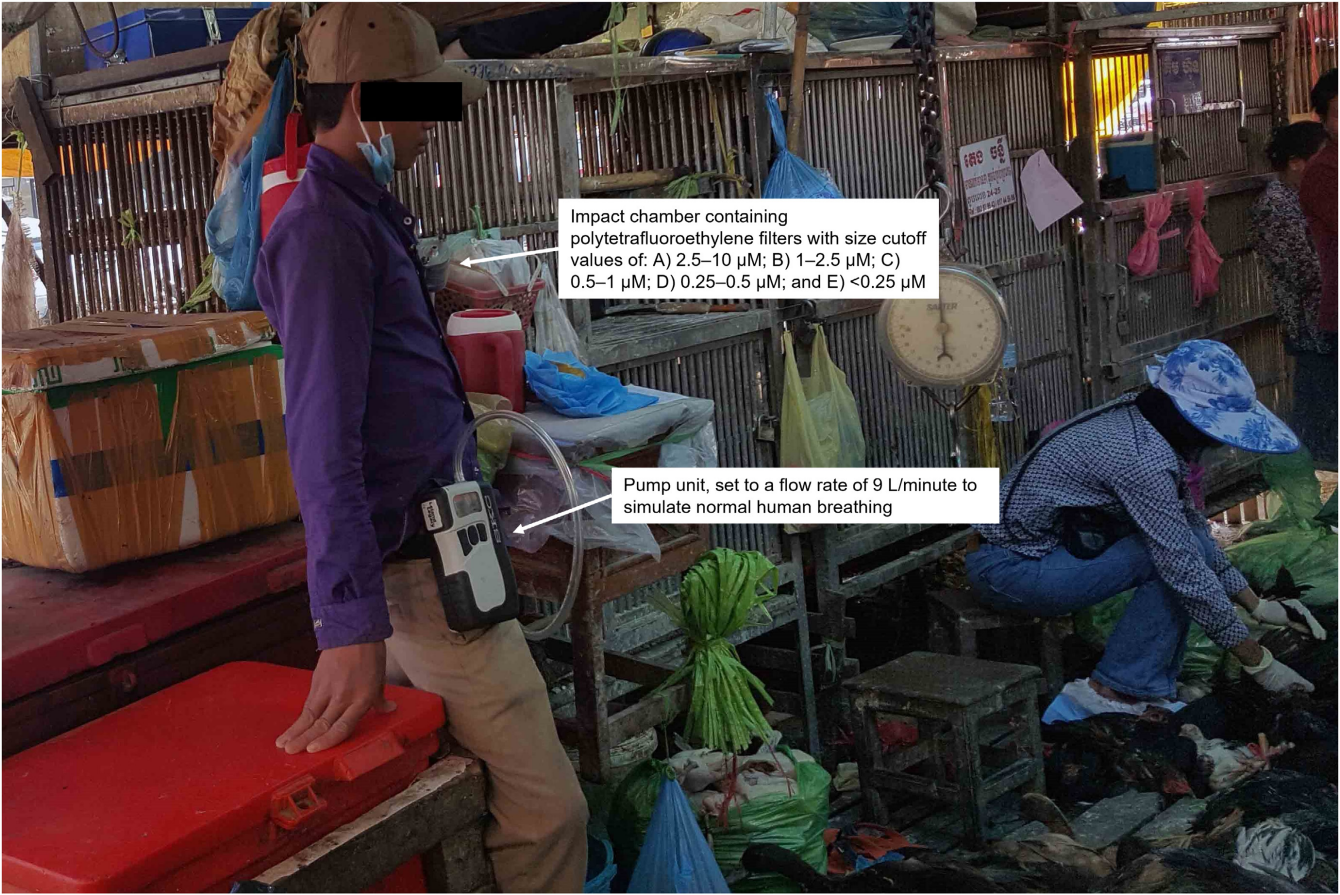
A live bird market vendor wearing a personal aerosol impact sampler during the study

Samples were collected from a central LBM in Phnom Penh, Cambodia, for five consecutive days during known periods of peak and low AIV circulation, February 22–26 and May 23–27, 2016, respectively. Oropharyngeal and cloacal swabs (pooled for each animal) were concurrently collected from four ducks and chickens selected randomly from throughout the LBM each day. Two 50 ml samples of carcass wash water (water used to wash poultry carcasses once slaughtered and defeathered) were also collected from two randomly selected containers in the market each day. To test the LBM air, two LBM vendors agreed to wear a Sioutas Personal Cascade Impactor Sampler (SKC Inc.) each day of collection for 30 min while continuing with their usual activities in the LBM (Figure 1). The pump unit (Leland Legacy pump: SKC Inc.) was operated at a flow rate of 9 L/min to simulate normal human breathing. The impact chamber contained five collection stages of polytetrafluoroethylene filters (SKC Inc.) with size cutoff values of (A) 2.5–10 μM; (B) 1–2.5 μM; (C) 0.5–1 μM; (D) 0.25–0.5 μM; and (E) <0.25 μM. These methods have been described previously for the detection of aerosolised influenza A/H3N2 in an indoor setting (Lednicky & Loeb, 2013). Following 30 min of wearing the impact sampler, the filter unit was immediately transported to the laboratory in an ice box to reduce inactivation of the viruses due to desiccation. Individual filters were then cut into three pieces, using sterile techniques, and placed in 3 ml of viral transport media (VTM). The VTM tube was vortexed to assist in removing material from the filters, split into three aliquots and stored at −80°C until required for testing.

All samples were processed according to protocols detailed previously (Horm et al., 2016; Horwood et al., 2018; Karlsson et al., 2019). Briefly, nucleic acids were extracted from all samples (processed air filters, wash water samples and chicken/duck swabs) using the QIAamp Viral RNA Mini Kit (QIAGEN), according to the manufacturer's instructions. Extracts were then tested for influenza matrix (M) gene, H5 (H5a and H5b primer sets), H7, and H9 by real‐time RT‐PCR. All assays were sourced from the International Reagent Resource (https://www.internationalreagentresource.org/). Samples that tested positive for influenza A virus were inoculated into specific pathogen‐free embryonated chicken eggs using standard methods (Horm et al., 2012).

Ethical approval for this study was granted by the Cambodian National Ethics Committee for Human Research (approval No. 051NECHR). Animal sampling was conducted by the National Animal Health and Production Research Institute (NAHPRI) under the direction of the General Directorate for Animal Health and Production, Cambodian Ministry of Agriculture, Forestry and Fisheries as part of routine disease surveillance activities.

## 3 RESULTS

Throughout the 2 weeks of collection, 20 LBM vendors (10 vendors per period) wore the air filter apparatus for 30 min while conducting their usual activities. AIV was detected by real‐time RT‐PCR in the filter apparatus significantly more frequently during the peak season in February (100%) compared with the low period in May (0%). Influenza A/H5 and A/H9 were detected in 40% and 50% of the air samples collected during the peak period of circulation, respectively (Table 1). No patterns were observed that suggested AIVs were detected more frequently in the larger or smaller pore size filters (Table S1).

**TABLE 1:** Summary results from samples collected from a Cambodian live bird market during peak (February) and low (May) periods of avian influenza circulation

	Detection and isolation of avian influenza viruses				
	M‐gene^a^	H5 (H5a/H5b)^a^	H7^a^	H9^a^	Isolation^b^
Peak period (Feb)					
Aerosol (*n* = 10)	10 (100%)	4 (40%)	0	5 (50%)	H5N1: 5 (50%); H9N2: 2 (20%)
Chicken (*n* = 20)	14 (70%)	6 (30%)	0 (0%)	6 (30%)	ND
Duck (*n* = 20)	15 (75%)	8 (40%)	0 (0%)	4 (20%)	ND
Wash water (*n* = 10)	10 (100%)	ND	ND	ND	ND
Low period (May)					
Aerosol (*n* = 10)	0 (0%)	0 (0%)	0 (0%)	0 (0%)	0 (0%)
Chicken (*n* = 20)	10 (50%)	0 (0%)	0 (0%)	6 (30%)	ND
Duck (*n* = 20)	3 (15%)	1 (5%)	0 (0%)	2 (10%)	ND
Wash water (*n* = 10)	10 (100%)	ND	ND	ND	ND

^a^ Avian influenza detection was conducted using real‐time RT‐PCR using assays sourced from the International Reagent Resource (https://www.internationalreagentresource.org/Home.aspx).

^b^ Isolation of avian influenza viruses was attempted using embryonated chicken eggs.

Live virus was recovered by egg‐based amplification from the air filter apparatus of 50% of participants from samples collected during peak AIV circulation (February). Influenza A/H5N1 was recovered from 5 (50%) of the participant samples collected during this period; and in 2 (20%) of these samples A/H9N2 was also concurrently isolated. Live virus was not isolated from any of the samples during the period of low circulation (May).

During the same 2 weeks of sampling (1 week during high and low AIV circulation, respectively), 40 chicken samples, 40 duck samples and 20 wash water samples were also collected. During February, AIV RNA was detected in 70%, 75%, and 100% of chicken, duck, and wash water samples, respectively. In May, AIV RNA was detected in 50%, 15% and 100% of chicken, duck and wash water samples, respectively. A/H5 subtype RNA was detected in 30% of chicken and 40% of duck samples during the peak period, compared with 0% and 5% during the low period, respectively. A/H9 subtype RNA was detected in 30% of chicken and 20% of duck samples during the peak period, compared with 30% and 10% during the low period, respectively. Influenza A/H7 RNA was not detected in any of the air, bird, or environmental samples throughout the study (Table 1).

**FIGURE 2:**
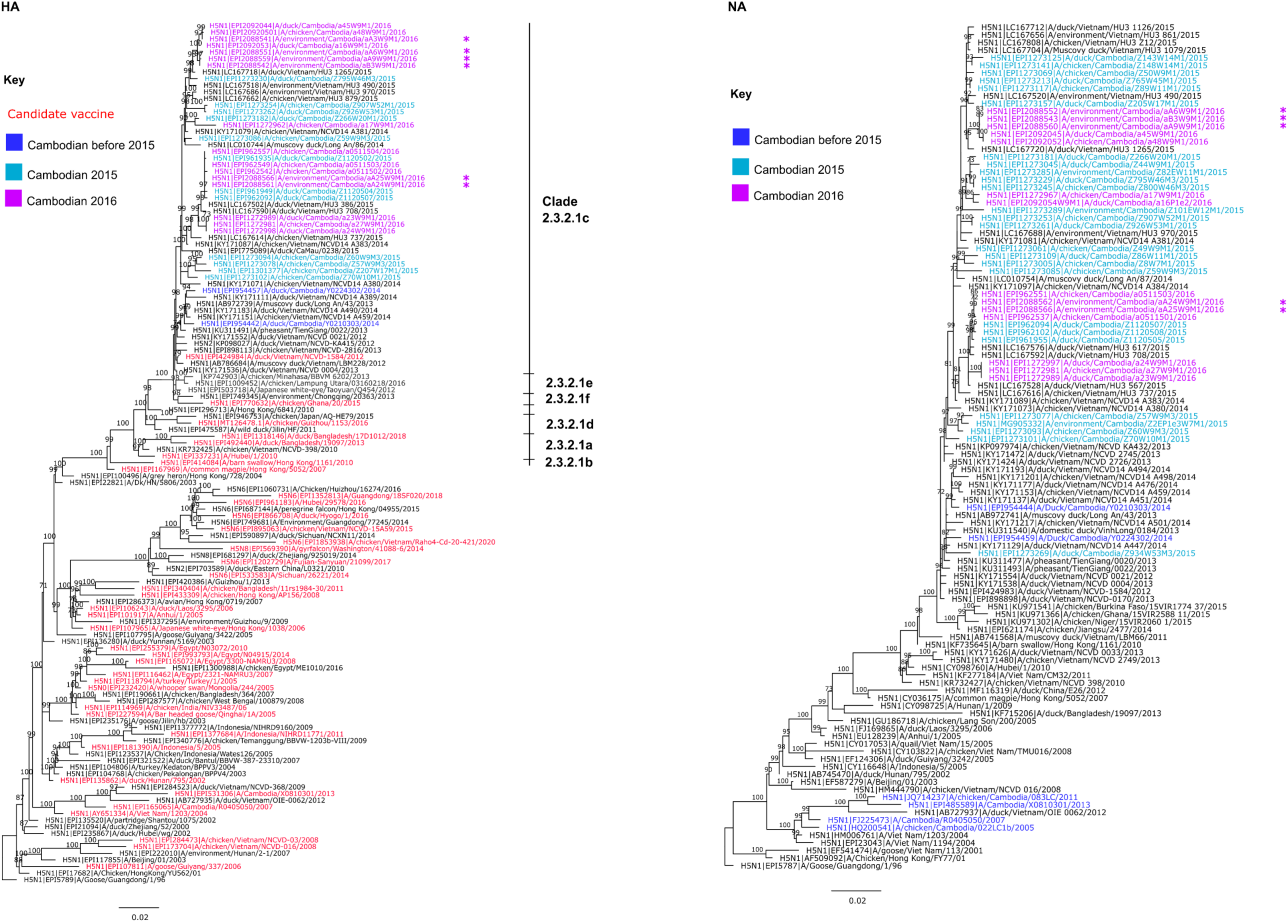
Maximum likelihood phylogeny of the hemagglutinin (HA) and neuraminidase (NA) genes of A/H5N1 viruses detected in air samples in a Cambodian live bird market, 2016. (A) HA and (B) NA of A/H5N1 clade 2.3.2.1c viruses in air and poultry samples. Trees were generated with IQ‐tree using the TVM + V + I + G4 and GTR + F + G4 model, respectively. Phylogenetic support was estimated using 1000 ultrafast bootstrap replicates. Cambodian isolates are shown before 2015 (dark blue), during 2015 (teal) and in 2016 (pink). Air samples from this study are indicated with a pink star. Candidate vaccine viruses are indicated in red. Bootstrap values are indicated next to significant nodes

Sequence analysis of the hemagglutinin (HA) and neuraminidase (NA) genes of several A/H5N1 isolates using Sanger methodology revealed the influenza A/H5N1 viruses to be typical of other clade 2.3.2.1c viruses circulating in LBMs during 2015–2016 (Suttie et al., 2019). Comparison of HA and NA sequences from air and poultry samples shows direct correlation, indicating that poultry within the LBM were the source of the viruses in the air (Figure 2).

## 4 DISCUSSION

Live bird markets have been identified as key locations for zoonotic infections with AIVs such as A/H5N1 and A/H7N9 (Cowling et al., 2013). In these environments, viruses may be aerosolised by a number of means including defeathering poultry (manual or mechanized) or suspended in dust from faeces or feather follicles. A range of influenza viruses have previously been detected through aerosol collection from LBMs in Asia, including A/H7N9, A/H9N2 and A/H5N6 (Bui et al., 2019; Wang et al., 2020; Wu et al., 2017, 2018; Zeng et al., 2017; Zhou et al., 2016). The high rate of detection during the peak period of circulation is very concerning for the risk of infection in susceptible individuals.

Avian influenza virus detection in poultry and wash water correlated with previous studies, with considerably higher rates of AIV positivity in February compared with May. This study demonstrates the increased risk of aerosol exposure of LBM workers to viable AIVs during periods of high circulation, and highlights the need for more research and risk‐reduction interventions during these high‐risk periods. Novel methods for monitoring pathogen circulation in high‐risk interfaces, such as environmental sampling, need to be further explored to identify improved approaches to surveil for pandemic threats. Environmental sampling presents an attractive alternative (or complement) to traditional sampling of individual animals. The collection of environmental samples is non‐invasive, non‐intrusive and is well accepted by market vendors. These approaches have the potential to provide longitudinal monitoring of live animal markets and are conducive to automation, potentially with the use of metagenomic analysis for unbiased monitoring of emerging pathogens. Further studies are needed to determine the optimal sampling frames to ensure high sensitivity and low cost.

## CONFLICT OF INTEREST

The authors declare no conflicts of interest.

## Supporting information


Table S1

## Data Availability

The data that supports the findings of this study are available in the supplementary material of this article
